# Differential Lung Ventilation Under the Veno-Arterial Extracorporeal Membrane Oxygenation Assistance in a Patient With a Giant Thoracic Tumor: A Case Report

**DOI:** 10.7759/cureus.58773

**Published:** 2024-04-22

**Authors:** Sumiko Sasaki, Kazunobu Une, Mai Nishina, Minoru Yamaki, Ryuichi Nakanuno

**Affiliations:** 1 Department of Emergency Medicine, Onomichi General Hospital, Hiroshima, JPN; 2 Department of Respiratory Surgery, Onomichi General Hospital, Hiroshima, JPN; 3 Department of Anesthesiology, Onomichi General Hospital, Hiroshima, JPN

**Keywords:** thoracic tumor, extracorporeal circulation, differential lung ventilation, bleeding risk, obstetric difficult airway management

## Abstract

Airway compression resulting from thoracic tumors requires evaluation of the possibility of fatal ventilation failure when securing the airway.

A woman presenting with a thoracic mass on the right side causing airway compression at the level of tracheal bifurcation required tumor removal to alleviate the compression; however, securing the airway proved challenging. Furthermore, differential lung ventilation was necessary for surgical management. We planned to secure the airway and manage breathing with the assistance of veno-arterial extracorporeal membrane oxygenation (V-A ECMO) through an interdisciplinary conference and proceeded according to the plan. The intended tracheal tube could be placed, differential lung ventilation was initiated, and the ECMO was removed. The surgical procedure was carried out.

In patients presenting with airway stenosis, the possibility of difficulty in securing the airway and ventilation should be assessed in advance. Creating a detailed treatment plan before surgery is recommended.

## Introduction

In cases of airway compression owing to a large thoracic tumor, it is necessary to evaluate the possibility of fatal ventilation failure when securing the airway. Furthermore, differential lung ventilation may be necessary for surgical management. In such cases, airway compression may worsen due to drug administration or position changes during anesthesia induction, making it difficult to secure the airway and potentially causing serious disorders. Therefore, sufficient preliminary evaluation and preoperative planning with multiple departments is required to ensure successful anesthesia management and surgery.

## Case presentation

A female in her 50s (height: 158 cm; weight: 40 kg) presented with a large thoracic mass (135×175 mm) on the right side causing airway compression at the level of the tracheal bifurcation.

**Figure 1:**
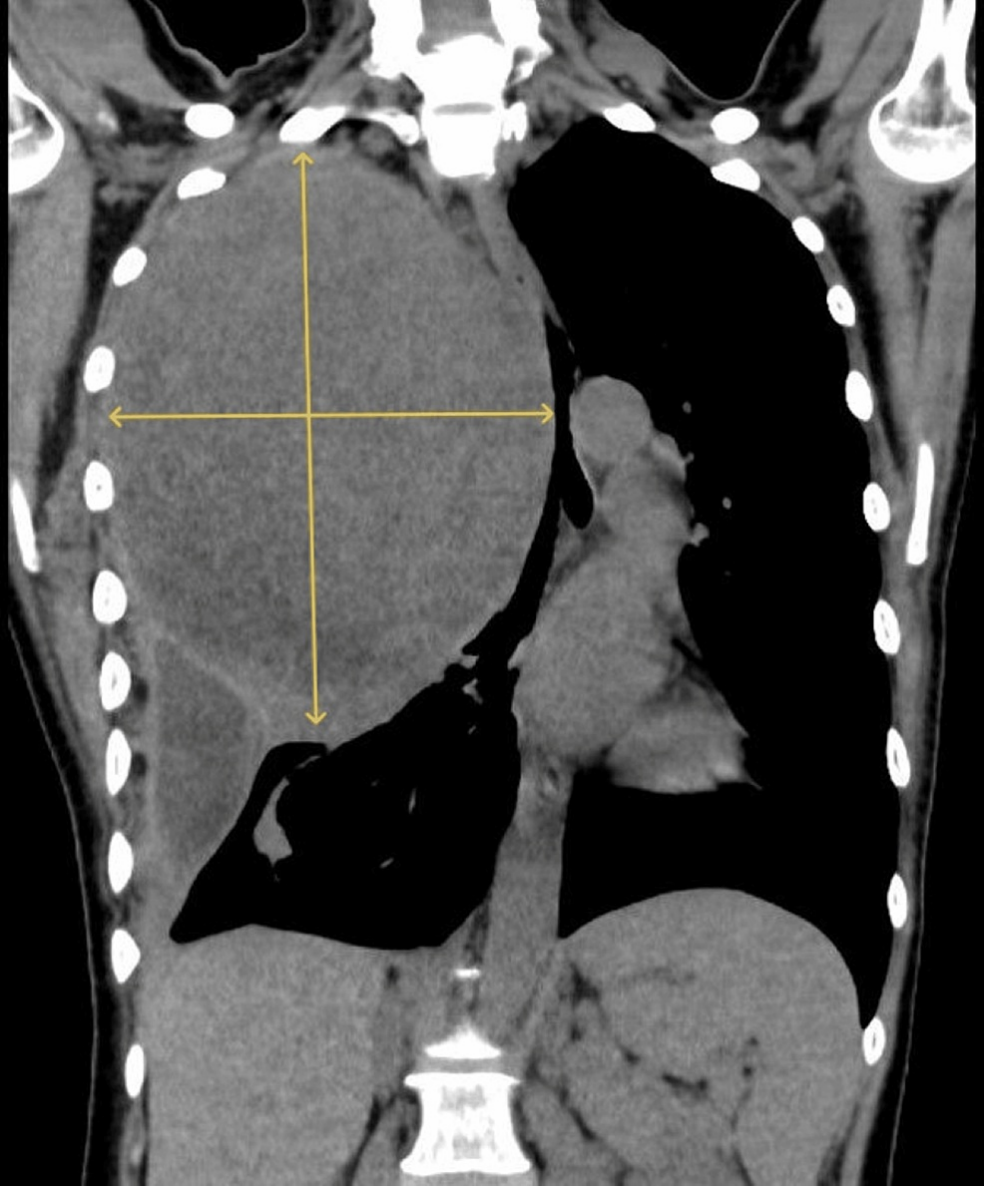
Contrast-enhanced CT of the chest at admission (coronal image) Three-dimensional CT of the trachea and bronchi. The right lung and trachea are compressed by a large mass (a chest tumor measuring 135x175mm). CT, computed tomography

**Figure 2:**
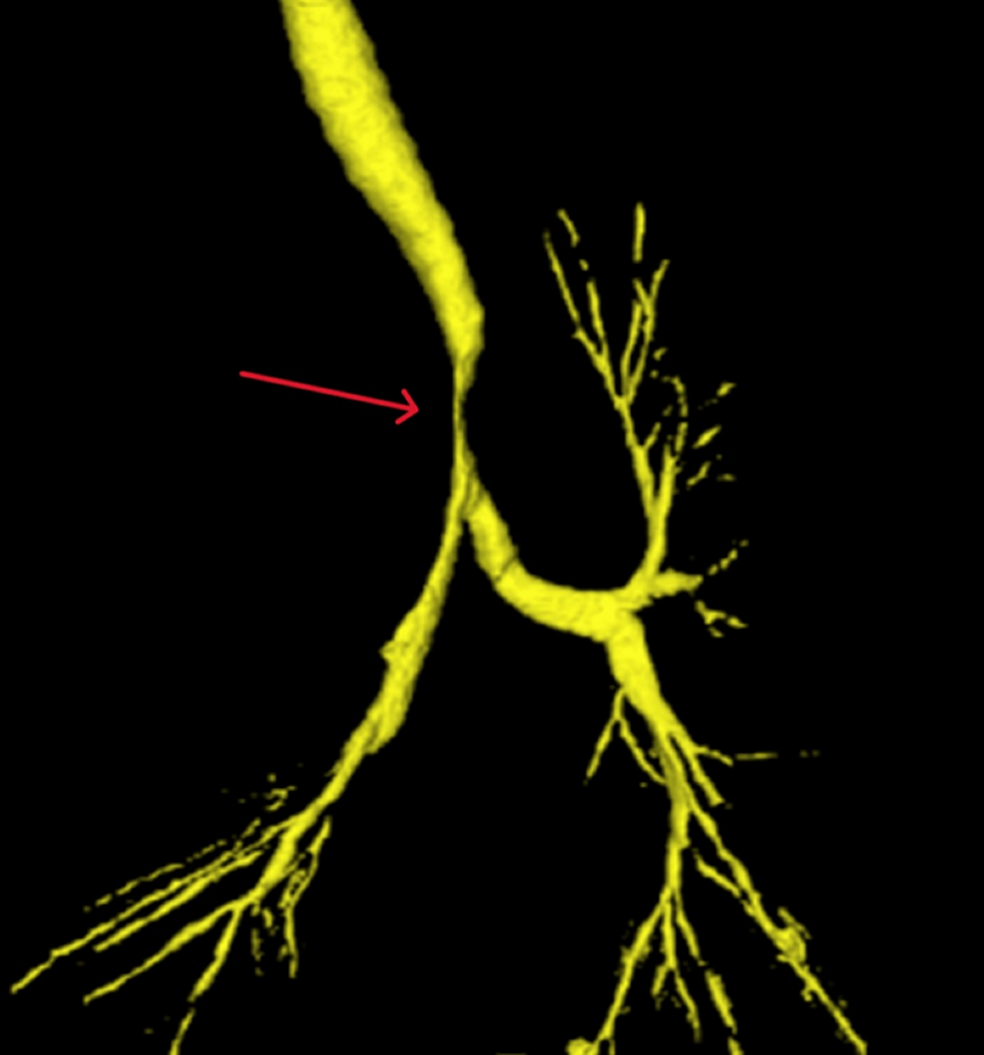
Three-dimensional CT of trachea and bronchi The red arrow indicates the most narrowed part of the trachea. The right lung and trachea are compressed by a large mass.

Two months before admission, the patient started experiencing dyspnea while lying down, which gradually worsened over time. On the day of the emergency visit to our hospital, the patient experienced difficulty breathing in the morning, and her symptoms persisted without improvement. Although the patient was able to walk, auscultation revealed a loss of breath sounds in her right chest, and a chest computed tomography (CT) scan revealed a large tumor in her right chest. She exhibited severe deviation of the mediastinum, including the trachea (Figures 1-2); therefore, we determined that the patient was at a high risk of airway obstruction. Consequently, she was admitted on an emergency basis.

Her Glasgow Coma Scale score was 15-E4V5M6, blood pressure was 126/78 mmHg, heart rate was 92 beats/min, respiratory rate was 22/min, and SpO_2_ was 88% on room air. The SpO_2_ increased to 96% with the administration of 2 L/min oxygen.

Although surgery was deemed necessary to relieve the airway compression, there was concern that securing the airway and managing breathing would be difficult during anesthesia induction. The narrowest part of the trachea was 15 mm above the tracheal carina and was severely narrowed by 3.2 mm from side to side. After considering the risk associated with anesthesia induction, we determined that securing the airway and performing differential lung ventilation, following assurance of oxygenation with extracorporeal membrane oxygenation (ECMO), would be safe. Regarding the selection of blood vessels for ECMO insertion, it was assumed that cannulation would be difficult because of the compression of the arteriovenous artery in the right neck by the tumor. Therefore, we decided to insert a delivery blood vessel from the thigh. In addition, we believed that with veno-venous ECMO (V-V ECMO), in which blood is transfused and extracted through the left and right femoral veins, there could be difficulty in maintaining a sufficient flow rate due to changes in body position. Consequently, the patient was scheduled to undergo V-A ECMO.

**Figure 3:**
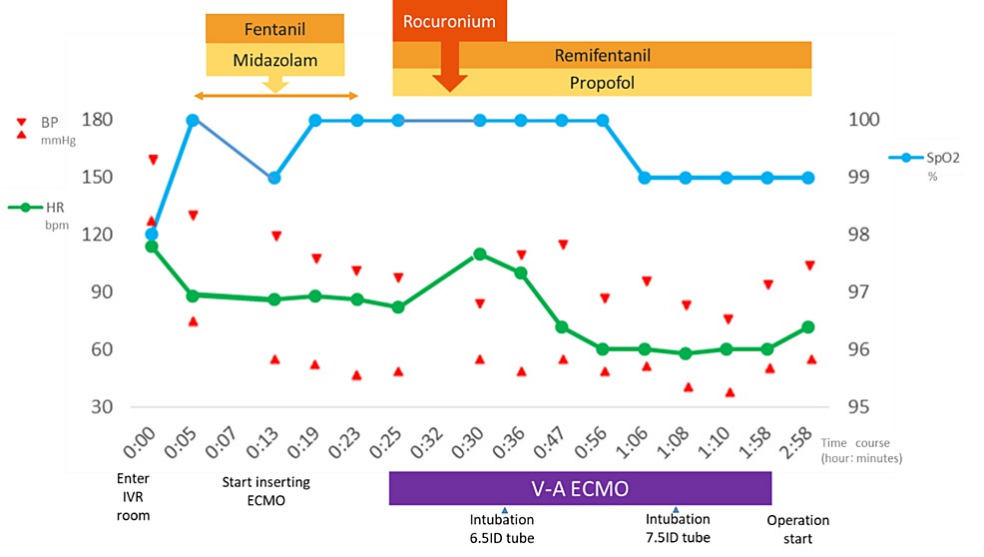
Preoperative vital signs; we inserted the V-A ECMO and intubated the trachea while the patient was semi-awake V-A ECMO, veno-arterial extracorporeal membrane oxygenation

On the fourth day of hospitalization, the right femoral artery and vein were percutaneously punctured under local anesthesia in the angiography room, and 16-Fr and 22-Fr tubes were inserted to establish extracorporeal circulation (Figure 3).

In anticipation of a prolonged surgery with ECMO management, if difficulties arose in securing the airway, continuous administration of heparin at 400 units/h was initiated after an intravenous injection of 5000 units of heparin. The activated clotting time (ACT) immediately after heparin introduction was 184 s. Next, sedation and muscle relaxation were performed and a tracheal tube with an internal diameter (ID) of 6.5 mm was inserted using a video laryngoscope, confirming that the tip passed through the narrowest part. There was no resistance during insertion and no bleeding; therefore, we replaced the tracheal tube with another one that had an ID of 7.5 mm and confirmed that the left lung was sufficiently ventilated. The patient was taken off ECMO before beginning surgery, and a total of 60 mg of protamine sulfate was administered to normalize the ACT. Intraoperative differential lung ventilation was maintained using a type S TCB bronchial blocker tube from Phycon🄬 for about 3.5 hours intermittently, that is, for 37 minutes during tumor removal and then for 3 hours and 7 minutes to stop bleeding from the chest wall. During the surgery, no hypoxemia with a SpO_2_ below 90% was observed. The mass, measuring 150×15 mm and weighing 1480 g, was surgically removed. The pathological diagnosis was a schwannoma. The bleeding continued from the chest wall after the resection, and a large amount of blood was transfused, with a total blood loss of 7800 mL. Postoperatively, the patient was admitted to the intensive care unit, and her progress was uneventful. On chest radiographs, the tracheal exclusion findings disappeared, and she was extubated the day after the surgery and began rehabilitation. She continued to progress uneventfully and was discharged on the 20th day of hospitalization. 

## Discussion

In recent years, several reports have suggested that the use of ECMO should be considered in cases of upper airway stenosis due to neck tumors, such as thyroid tumors, and anesthesia management under ECMO assistance has been reported [1,2]. The difference between this case and previously reported cases was the presence of central intrathoracic airway stenosis centered at the tracheal carina. The plan was to surgically remove the tumor, but it was assessed beforehand ensuring that there was a high possibility of securing the airway and managing breathing during anesthesia induction. Therefore, we held discussions with doctors from multiple departments, mainly respiratory surgery, the primary department involved, and shared information with operating room nurses and clinical engineers.

Concerning the selection of a tracheal tube, we determined that a double-lumen tube would have a high risk of insertion failure or bleeding, considering the degree of bronchial stenosis. We decided to use a bronchial blocker tube for differential lung ventilation. Also, an ID of 7.5 mm was considered optimal to effectively navigate the narrowest part, located just above the tracheal carina, and to facilitate differential lung ventilation. We developed a step-by-step plan for the safe insertion of the tracheal tube. First, we assumed the length of the tube necessary to pass through the narrowest part of the trachea and to position the tip directly above the carina; we found that the tracheal tube should have an ID of at least 6.5 mm for a diameter of 3.2 mm at the narrowest part. Therefore, our first goal was to insert a tracheal tube with an ID of 6.5 mm across the narrowest point. We refrained from bronchoscopic assistance owing to the risk of airway bleeding. To secure the airway in this case, we determined that tracheal intubation with ECMO assistance was the safer option. Additionally, if tracheal intubation proved impossible, even with a tracheal tube of the smallest diameter, we would perform the surgery with ECMO assistance. If it was possible to intubate with a tracheal tube with an ID of 6.5 mm as planned, we planned to switch to a tracheal tube with an ID of 7.5 mm, considering bleeding and resistance during insertion. Following this, we would remove the ECMO and proceed with the surgery under differential lung ventilation. This was the final goal of this treatment.

Regarding the selection of an approaching vessel for ECMO introduction, it was assumed that insertion from the right neck would be challenging due to vessel compression by the tumor. Therefore, the femoral artery and vein were selected. We considered V-V ECMO, which pumps blood into and out of the femoral vein. However, if we were to conduct surgery under ECMO management, maintaining an appropriate flow rate might prove challenging because of changes in the patient's position. Therefore, we decided to introduce V-A ECMO via the femoral artery and vein. However, in this case, there was no problem with cardiac function. V-A ECMO may cause differential ischemia, in which deoxygenated blood perfuses the coronary arteries and intracranial blood vessels when ventilation is stopped, causing myocardial and cerebral ischemia [2]. Referring to the report by Fujii et al., connecting closed soft reservoirs to the blood supply and removal sides of the circuit allows for blood storage in the reservoirs, thereby enabling the management of spontaneous cardiac output [3]. We decided to respond by decreasing the amount and increasing the retrograde blood flow.

It has been reported that the incidence of ECMO-related complications is lower when the duration of ECMO is shorter [4]. Therefore, to minimize bleeding, it is recommended to promptly remove the ECMO and perform surgery after normalizing the ACT. In this case, heparin was managed by adjusting the ACT value to 1.5 times the reference value, following the recommendations in the ELSO guidelines, and a total of 60 mg of protamine sulfate was administered to normalize the ACT [5]. It is crucial to remove the ECMO whenever possible and to sufficiently reverse anticoagulants when performing surgical treatment.

## Conclusions

Patients presenting with airway stenosis, regardless of upper airway stenosis, should be pre-evaluated for potential difficulties with airway management and ventilation. In thoracic surgery, there is a high possibility that cases will occur that require advanced anesthesia management such as isolated lung ventilation. We recommend creating a detailed treatment plan, including invasive procedures such as ECMO, before surgery. Additionally, when using ECMO, certain measures should be taken, such as early withdrawal from ECMO and monitoring of coagulation ability, to avoid bleeding complications during surgery as much as possible.
